# Supplementary material for: End Sequence Analysis Toolkit (ESAT) expands the extractable information from single-cell RNA-seq data
Source: Genome Res. 2016 Oct;26(10):1397–410. doi: 10.1101/gr.207902.116 (PMC5052061; doi:10.1101/gr.207902.116)
Supplement: Supplemental Material [file supp_gr.207902.116_Supp_Materials.tar.gz › ESAT-esat_v0.1/doc/html/classbroad_1_1pda_1_1enrichment_1_1_enrichment_utils.html]

ESAT: broad.pda.enrichment.EnrichmentUtils Class Reference


ESAT


- Main Page
- Related Pages
- Packages
- Classes
- Files

- Class List
- Class Index
- Class Hierarchy
- Class Members

All Classes Namespaces Files Functions Variables Enumerator Pages

- broad
- pda
- enrichment
- EnrichmentUtils

Static Public Member Functions |
List of all members

broad.pda.enrichment.EnrichmentUtils Class Reference

|  |  |
| --- | --- |
| Static Public Member Functions | |
| static SortedMap< String, File > | findAnnotationFiles (File dir, String[] fileExt) |
|  | |
| static SortedMap< String, File > | findAnnotationFiles (File dir, String[] fileExt, String basename) |
|  | |
| static SortedMap< String, File > | findAnnotationFiles (File dir, String[] fileExt, String basename, SortedMap< String, File > map) |
|  | |

## Member Function Documentation

|  |  |  |  |
| --- | --- | --- | --- |
| static SortedMap<String, File> broad.pda.enrichment.EnrichmentUtils.findAnnotationFiles | ( | File | *dir*, |
|  |  | String[] | *fileExt* |
|  | ) |  |  |

static

Recursively visits directories to find genomic annotation files.

Parameters
:   |  |  |
    | --- | --- |
    | dir | Base directory |
    | fileExt | List of valid genomic annotation file extensions |

Returns
:   Map of genomic annotation names and the corresponding files.

Here is the caller graph for this function:

|  |  |  |  |
| --- | --- | --- | --- |
| static SortedMap<String, File> broad.pda.enrichment.EnrichmentUtils.findAnnotationFiles | ( | File | *dir*, |
|  |  | String[] | *fileExt*, |
|  |  | String | *basename* |
|  | ) |  |  |

static

Here is the call graph for this function:

|  |  |  |  |
| --- | --- | --- | --- |
| static SortedMap<String, File> broad.pda.enrichment.EnrichmentUtils.findAnnotationFiles | ( | File | *dir*, |
|  |  | String[] | *fileExt*, |
|  |  | String | *basename*, |
|  |  | SortedMap< String, File > | *map* |
|  | ) |  |  |

static

Here is the call graph for this function:

---

The documentation for this class was generated from the following file:

- java/broad/pda/enrichment/EnrichmentUtils.java


---

Generated on Fri Aug 15 2014 15:10:59 for ESAT by  

 1.8.7
